# Functional characterization of naturally-occurring constitutively activating/inactivating mutations in equine follicle-stimulating hormone receptor

**DOI:** 10.5713/ab.21.0246

**Published:** 2021-08-24

**Authors:** Munkhzaya Byambaragchaa, Tae-Young Ahn, Seung-Hee Choi, Myung-Hwa Kang, Kwan-Sik Min

**Affiliations:** 1 Animal Biotechnology, Graduate School of Future Convergence Technology, Hankyong National University, Ansung 17579, Korea; 2 Department of Food Science and Nutrition, Hoseo University, Asan 31499, Korea; 3 School of Animal Life Convergence Science, Institute of Genetic Engineering, Hankyong National University, Ansung 17579, Korea

**Keywords:** Equine Follicle-stimulating Hormone Receptor, Constitutive Activation, Allelic Variant Mutation, Inactivating Mutation, cAMP Responses

## Abstract

**Objective:**

Follicle-stimulating hormone (FSH) is the central hormone involved in mammalian reproduction, maturation at puberty, and gamete production that mediates its function by control of follicle growth and function. The present study investigated the mutations involved in the regulation of FSH receptor (FSHR) activation.

**Methods:**

We analyzed seven naturally-occurring mutations that were previously reported in human FSHR (hFSHR), in the context of equine FSHR (eFSHR); these include one constitutively activation variant, one allelic variant, and five inactivating variants. These mutations were introduced into wild-type eFSHR (eFSHR-wt) sequence to generate mutants that were designated as eFSHR-D566G, -A306T, -A189V, -N191I, -R572C, -A574V, and -R633H. Mutants were transfected into PathHunter EA-parental CHO-K1 cells expressing β-arrestin. The biological function of mutants was analyzed by quantitating cAMP accumulation in cells incubated with increasing concentrations of FSH.

**Results:**

Cells expressing eFSHR-D566G exhibited an 8.6-fold increase in basal cAMP response, as compared to that in eFSHR-wt. The allelic variation mutant eFSHR-A306T was not found to affect the basal cAMP response or half maximal effective concentration (EC_50_) levels. On the other hand, eFSHR-D566G and eFSHR-A306T displayed a 1.5- and 1.4-fold increase in the maximal response, respectively. Signal transduction was found to be completely impaired in case of the inactivating mutants eFSHR-A189V, -R572C, and -A574V. When compared with eFSHR-wt, eFSHR-N191I displayed a 5.4-fold decrease in the EC_50_ levels (3,910 ng/mL) and a 2.3-fold decrease in the maximal response. In contrast, cells expressing eFSHR-R633H displayed in a similar manner to that of the cells expressing the eFSHR-wt on signal transduction and maximal response.

**Conclusion:**

The activating mutant eFSHR-D566G greatly enhanced the signal transduction in response to FSH, in the absence of agonist treatment. We suggest that the state of activation of the eFSHR can modulate its basal cAMP accumulation.

## INTRODUCTION

Follicle-stimulating hormone receptor (FSHR) belongs to the family of class A G-protein coupled receptors (GPCR)s, which includes luteotropin/choriogonadotropin receptor (LH/CGR) and thyroid-stimulating hormone receptor (TSHR). These receptors have a large extracellular domain for ligand binding, seven transmembrane helical domains, and a cytoplasmic tail with phosphorylation sites. FSHR plays an important role on the surface of reproductive cells, including ovarian, testes tissues by binding to follicle-stimulating hormone (FSH) with high affinity. The receptor then uncouples from the G-receptor and the desensitized hormone-receptor complex gets internalized into the endosomes, following which most of the complex is recycled back to the cell surface and partially degraded in the lysosomes [1–5]. In human FSHR (hFSHR), many studies have reported the presence of naturally-occurring constitutively activating/inactivating mutations as well as induced mutations of residues, suggesting that the signal transduction has important effects in various aspects of receptor function [6–12].

The activating mutant of FSHR, hFSHR-D576G, was first reported in a 28-year old hypophysectomized male. The hFSHR-D567G mutant (equivalent to D566G in equine FSHR [eFSHR]) was found to display 3-fold higher basal cAMP production compared to wild-type FSHR (FSHR-wt) [8]. Thus, this suggests that this particular site is involved in the ligand-independent constitutive activation of FSHR. Located on the third intracytoplasmic loop, this mutation site is highly conserved in all glycoprotein hormone receptors [13]. The same mutation has been detected in TSHR in patients with thyroid adenoma and pseudoprecocious puberty [14,15]. The human LHR-D578G mutant, found in patient with the syndrome of familial male-limited precocious puberty, has been shown to display approximately 15- to 25-fold higher basal cAMP production in HEK 293 cells compared to hLHR-wt [16]. Thus, this activating mutant seems to play a pivotal role in the signal transduction through the receptor; specifically by increasing the basal cAMP accumulation in the absence of agonist-treatment.

The allelic variant, A307T in hFSHR (equivalent to A306T in eFSHR) was first reported in the extracellular domain following screening of the *FSHR* gene in patients with hypogonadotropic ovarian dysgenesis (ODG) [1,6], premature ovarian failure (POF) [17], women with hypogonadotropic hypogonadism, and in infertile men [13]. hFSHR-N680S, located in the intracellular domain, was identified in cases of ODG with a normal karyotype [6] and ovarian granulosa cell tumor [18]. Patients with ovarian juvenile granulosa cell tumors have also been found to harbor polymorphisms at the 307 and 680 amino residues [18,19]. However, there is no report whether these polymorphisms have important roles in FSH binding and signal transduction. Thus, the function of the naturally-occurring polymorphisms in the FSHRs needs to be elucidated in order to characterize the receptor and ligand.

In case of the inactivating mutation, a patient with ODG was found to harbor a transition from Ala to Val at position 189; this mutation, located in the extracellular domain of hFSHR is conserved within all glycoprotein hormone receptors and within FSHRs from different species [6,20]. Upon genotyping the A189V mutation of FSHR in 15 brothers from families with ODG, 4 were identified as wild-type homozygotes, 6 as heterozygotes, and 5 as homozygotes, suggesting that men do not show azoospermia or absolute infertility [21]. In addition, studies demonstrate that FSH is more important for female fertility than male fertility. This mutation was identified to be homozygous in all affected females, contributed to the disease phenotype, and impaired the signal transduction in transfected mouse Sertoli cells (MSC-1 cells). The Asn191Ile mutation, close to Ala189, was also observed in a heterozygous fertile woman showing normal ovarian function [8]. However, the cAMP response to FSH was found to be completely abolished in the *in vitro* signal transduction. This sequence involves a potential N-linked glycosylation site; in case of rat FSHR, this mutation (N173Q) has been shown to be important for proper folding of the hormone [22,23]. The sequence of five amino acids, from Aal189 to Thr193, including Asn191 residue, was found to be perfectly conserved in all glycoprotein hormone receptors, suggesting its important in the signal transduction through the receptor.

Two other inactivating mutations (Ile160Thr and Arg573Cys) were reported that in a woman with secondary amenorrhea who had very high FSH concentration in the plasma, despite normal ovarian size [24]. A previous study also identified two naturally-occurring mutations (Val514Ala and Ala575Val) in a woman with primary amenorrhea and ovarian hyperstimulation syndrome (OHSS); the study showed that a dose-dependent increase in cAMP levels upon FSH stimulation was not observed in case of the Ala575Val-expressing mutant [25]. Binding affinity and cAMP accumulation were barely detected in cells expressing the D224V mutant receptor, found in a patient with primary amenorrhea [26]. The mutation (R634H) located in the cytoplasmic tail of the receptor was first described in a case of spontaneous ovarian hyperstimulation syndrome (sOHSS). The R634H-expressing mutant was found to decrease cAMP production in response to FSH and markedly reduce cell surface expression [27]. Recently, several other inactivating mutations and polymorphisms have been reported: -29G>A identified in the promoter region by polymerase chain reaction (PCR) screening [28], -29G>A and A189V identified by PCR-restriction fragment length polymorphism [12], Ile418Ser in a patient with primary ovarian failure [29], Asp408Tyr identified by whole exome sequencing in the second transmembrane domain of FSHR [30], and a novel homozygous mutation (R59X) in exon 2 [31]. Thus, this indicates that the intracellular loop 3 and cytoplasmic tail region of FSHR play an important function in signal transduction. We also recently reported that the basal cAMP response of eelFSHR-D540G exhibited a 23-fold increase in the absence of agonist treatment [32].

Several studies on the characterization of naturally-occurring FSHR mutations have mainly focused on hFSHR. To date, nothing is known about the signal transduction of the naturally-occurring mutations in case of eFSHR. The goal of the present study is to determine whether the mutations that activate or inactivate hFSHR also affect eFSHR in the same way. Thus, naturally- occurring eFSHR mutations, including one constitutively activating, one allelic variant, and five inactivating mutations were generated by site-directed mutagenesis in eFSHR-wt and analyzed by *in vitro* functional characterization.

## MATERIALS AND METHODS

### Materials

The PCR kit including Ex Taq polymerase, restriction enzymes, and high-2 ligation solution were purchased from Takara (Shiga, Japan). The gels clean up system (purification kit) and cloning vector pGEMTeasy were from Promega (Madison, WI, USA). The oligonucleotides for PCR were synthesized by Genotech (Daejon, Korea). The plasmid extraction kit used was from GeneAll Biotechnology (Seoul, Korea). Opti-MEM I, penicillin & streptomycin, and L-glutamine were purchased from Gibco BRL (Grand Island, NY, USA). Fetal bovine serum (FBS) was from Hyclone Laboratories (Logan, UT, USA). Lipofectamine 2000 was purchased from Invitrogen Corporation (San Diego, CA, USA). Human FSH, AssayComplete medium, pCMV-ARMS1-PK2 expression vector, and PathHunter EA-parental CHO-K1 cells were from DiscoverX (Fremont, CA, USA). The homogeneous time-resolved fluorescence (HTRF) cAMP assay kit was purchased from Cisbio Bioassays (Codolet, France). QIAGEN Maxi plasmid kits were purchased from Qiagen Inc. (Hilden, Germany). All other reagents used were from Sigma-Aldrich (St. Louis, MO, USA) and Wako Pure Chemicals (Osaka, Japan).

### Site-directed mutagenesis and vector construction

**Figure 1:**
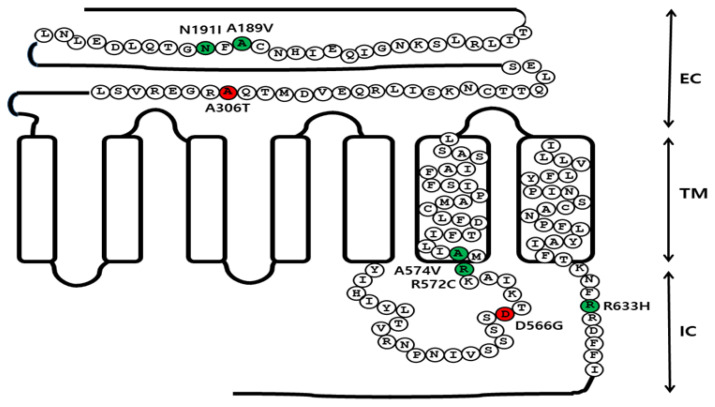
Schematic representation of the eFSHR structure. The location of the constitutively activating mutation (D566G), the five inactivating mutations (A189V, N191I, R572C, A574V, and R633H), and the allelic variant (A306T) are indicated in the structure of eFSHR. Red circles indicate the constitutively activating mutation and the allelic variant, while green circles indicate the inactivating mutations. Two inactivating mutations (A189V and N191I) and the allelic variant (A306T) are located in the EC domain. The activating mutation (D566G) is located in the third IC loop. The other two inactivating mutations (R572C and A574V) are located between the third IC loop region and the six TM. R633H is located in the intraplasmic tail region. eFSHR, equine follicle-stimulating hormone receptor; EC, extracellular domain; TM, transmembrane domain; IC, intracellular domain.

eFSHR cDNA was cloned using testicular and ovarian total RNA, as previously described [32]. eFSHR mutations were introduced into eFSHR-wt sequence using overlapping PCR mutagenesis, as previously described [32,33]. For generation of the constitutively activating mutation, Asp was substituted with Gly at residue 566 and the mutant was designated as eFSHR-D566G. NheI and SacI restriction enzyme sites were added at the 5′- and 3′-ends of eFSHR-wt sequence and the stop codon was not included in the C-terminal region of the template for cloning into pCMV-ARMS1-PK2 expression vector. The allelic variant mutant (eFSHR-A306T) and inactivating mutants (identified previously in hFSHR), were constructed by introducing the corresponding mutations at the positions 189 (A189V), 191 (N191I), 572 (R572C), 574 (A574V), and 633 (A633H) of eFSHR-cDNA, as shown in Figure 1. The PCR products were cloned into the pGEMTeasy cloning vector. After sequencing using automated DNA sequencing, the eFSHR-wt and mutant fragments were sub-cloned into the pCMV-ARMS1-PK2 expression vector using NheI and SacI restriction sites.

### Transient transfection

PathHunter EA-parental CHO-K1 cells (engineered to stably express a fusion protein of β-arrestin and the enzyme acceptor portion of β-galactosidase) were transiently transfected with the wild-type and mutant eFSHR constructs using lipofectamine, as previously described [33]. The cells were maintained in Assay Complete CHO-K1 culture medium supplemented with 10% FBS, 100 U/mL penicillin, and 100 μg/mL streptomycin. Cells were seeded into 6-well plates 18 h before transfection. After the cells were washed with Opti-MEM twice, the DNA-lipid complex was added to each well gently and the plate was incubated in a humidified atmosphere with 5% CO_2_ at 37°C. Assay complete medium supplemented (1 mL) with 20% FBS was added to each well after 5 h. The transfected cell medium was replaced with fresh culture medium 24 h post-transfection and the cells were cultured for one more day before cAMP assay was carried out.

### cAMP analysis by homogeneous time-resolved fluorescence

cAMP accumulation in PathHunter EA-parental CHO-K1 cells was measured using a cAMP Dynamic 2 competitive immunoassay kit (Cisbio Bioassays, France). Cells transfected with wild-type and mutant eFSHR-expressing constructs were suspended in a 0.5 mM 3-isobutyl-1-methylxanthine solution to prevent cAMP degradation. The cells were then seeded into 384-well white plates (Thermo Scientific, Waltham, MA, USA) at a density of 10^4^ cells/well, and treated with ligands (human FSH from DiscoverX, USA) in a dose-dependent manner for 30 min at room temperature. Following this, 5 μL dye d2-labeled cAMP solution and 5 μL Eu3+Cryptate-conjugated anti-cAMP antibody solution were added consistently to each assay well. After incubation for 1 h at room temperature, the fluorescent signal readout was carried out at 665 nm and 620 nm using a HTRF compatible Artemis K-101 microplate reader (Kyoritsu Radio, Minato-Ku, Japan). The results were calculated from the 665 nm/620 nm ratio and are expressed Delta F% (cAMP inhibition), according to the following equation:


Delta F%=(standard or sample ratio-mock transfection)×100/mock transfection

The cAMP concentrations for the Delta F% values were calculated using Prism software (GraphPad Software, Inc, La Jolla, CA, USA).

### Data analysis

The sequences were compared using Multalin interface-multiple sequence alignment tool. Each dose dependent data was analyzed from experiments performed in duplicate. The data of cAMP levels in all the transfected cells were subtracted from those in the mock-transfected cells. GraphPad Prism 6.0. was used to evaluate the differences between samples using one-way analysis of variance, followed by Turkey’s multiple comparison tests. A p-value of <0.05 was taken to indicate a significant between the groups.

## RESULTS

### Generation of substitution point mutants of eFSHR

In order to identity the constitutively activating/inactivation mutations and allelic variants that contribute to the effects in respect to FSH-induced signal transduction, individual mutants were constructed by replacing each residue in eFSHR-wt with the corresponding variant residues (A189V, N191I, A306T, D566G, R572C, R574V, and R633H) (Figure 1). These mutations have previously been identified as constitutively activating, inactivating, and allelic variant polymorphisms in hFSHR. Most of the selected mutations are conserved in all the glycoprotein hormone receptors, including FSHR, LHR, and TSHR. The identity of each mutant was confirmed, followed by sub-cloning into pCMV-ARMS1-PK2 and transfection into PathHunter EA-parental CHO-K1 cells expressing β-arrestin. These cells have been engineered to stably express the enzyme acceptor-tagged β-arrestin fusion protein [34]. This system can be used to detect ligand-induced activation of FSHR independent of G-protein coupling, by the co-expression of a ProLink-tagged GPCR.

### Signaling of constitutively activating and allelic variant mutants

**Figure 2:**
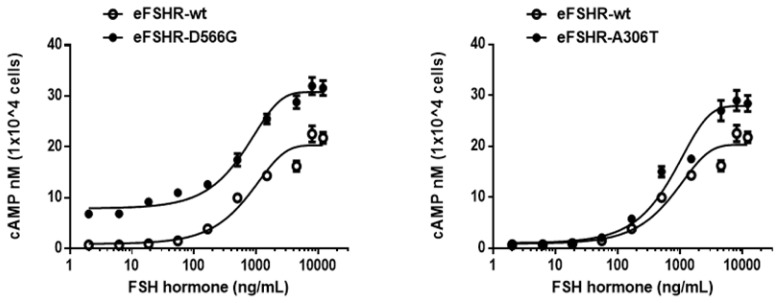
Total cAMP levels stimulated by FSH in PathHunter EA-parental CHO-K1 cells transfected with constitutively active and allelic variant eFSHR mutants. PathHunter EA-parental CHO-K1 cells transiently transfected with wild-type and mutant (D566G and A306T) eFSHRs were stimulated with FSH in a medium containing 0.5 mM 3-isobutyl-1-methyl xanthine for 30 min. cAMP production was detected using homogeneous time-resolved fluorescence assay. The cAMP accumulation is represented as Delta F%. cAMP concentration was recalculated and presented using GraphPad Prism software. The results of the mock-transfected cells subtracted from each data set (see Methods). Each point represents the average±standard error of the mean of triplicate experiments. The mean data were fitted to the equation for a one-phase exponential decay curve. The blank circles were the same curves of wild-type receptor. FSH, equine follicle-stimulating hormone; eFSHR, equine follicle-stimulating hormone receptor.

Cells transfected with eFSHR-wt DNA exhibited an increased production of cAMP in response to a high concentration of the FSH agonist. The half maximal effective concentration (EC_50_) of the eFSH-stimulated cAMP response was approximately 765.1 ng/mL (Figure 2). The basal and Rmax cAMP responses were 0.9 and 20.3 nM/10^4^ cells, respectively. The D566G mutation induced constitutive activation of eFSHR, as demonstrated by an 8.6-fold increase in the cAMP response, which was detected in the basal condition without the presence of the agonist. The maximal cAMP response elicited by the activating mutant was approximately 1.5-fold that of the wild-type, as shown in Table 1. cAMP production in the absence of agonist treatment indicates that the eFSHR-D566G mutant is constitutively active. Thus, cells expressing the mutation at this site show with a similar dose-dependent increase in cAMP response as the wild-type cells, but a higher basal cAMP accumulation, regardless of species, including fish [32].

Polymorphic variant screening identified allelic variants at two positions (A307T and N680S) in infertile men and POF patients. Of the two of them, we characterized the eFSHR-A306T mutant (located in the extracellular domain) *in vitro* in transiently transfected PathHunter EA-parental CHO-K1 cells. As shown in Figure 2, cAMP production in response to increasing concentrations of FSH was found to be similar in the eFSHR-A306T mutant-expressing and eFSHR-wt-expressing cells. The Rmax response was 1.4-fold higher in eFSHR-A306T-transfected cells, as compared to eFSHR-wt-transfected cells (Table 1). However, the EC_50_ level of the eFSHR-A306T allelic mutant (751.2 ng/mL) was similar to that of the wild-type receptor (765.1 ng/mL). Thus, in terms of cAMP response induced by FSH agonist treatment, the eFSHR-A306T allelic mutant showed no difference from eFSHR-wt.

### FSH-stimulated cAMP accumulation in cell lines expressing inactivating mutants

**Figure 3:**
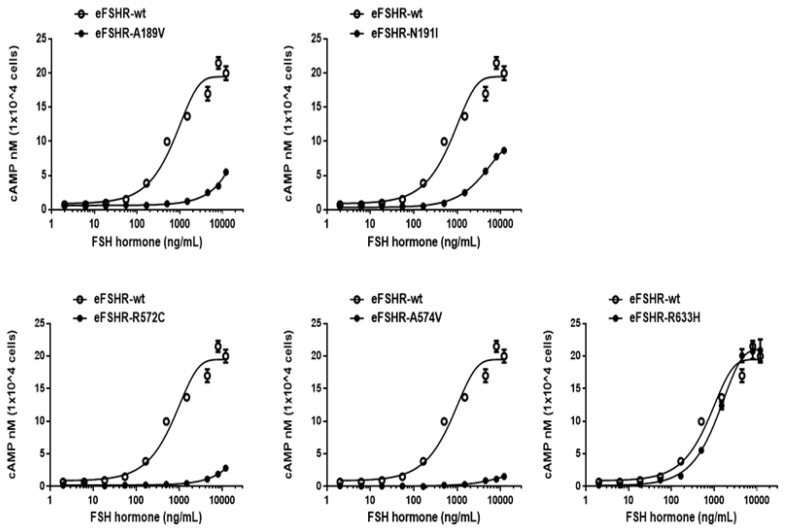
cAMP production, stimulated by FSH treatment, in PathHunter EA-parental CHO-K1 cells transfected with inactivating eFSHR mutants. PathHunter EA-parental CHO-K1 cells transiently transfected with wild-type eFSHR and eFSHR inactivating mutants (eFSHR-A189V, -N191I, -R572C, -A574V, and -R633H) were stimulated with FSH for 30 min. Total cAMP accumulation was analyzed using a homogeneous time-resolved fluorescence assay. The empty circles denote wild-type eFSHR and black circles denote the mutants. The values for the mock-transfected cells subtracted from each data point. Each point represents the average±standard error of the mean of triplicate experiments. The mean data were fitted to the equation for a one-phase exponential decay curve. FSH, equine follicle-stimulating hormone; eFSHR, equine follicle-stimulating hormone receptor.

The ability of the mutant eFSHRs to transduce FSH was measured by quantitating cAMP accumulation in cells incubated with increasing concentrations of FSH (Figure 3; Table 2). As shown in Table 2, there was a profound effect on the FSH responsiveness of eFSHR-A189V, -N191I, -R572C, and -A574V. The maximal response of cells transfected with these mutants was shown to be greatly reduced when compared to the equine wild-type control receptor.

Mutants harboring the eFSHR-R572C and -A574V variants were completely ineffective in cAMP accumulation, despite the high concentration of FSH. There was a 3.5- and 2.2-fold reduction in the maximal response of the other two mutants, eFSHR-A189V and -N191I, when compared to that of the wild-type control.

**Figure 4:**
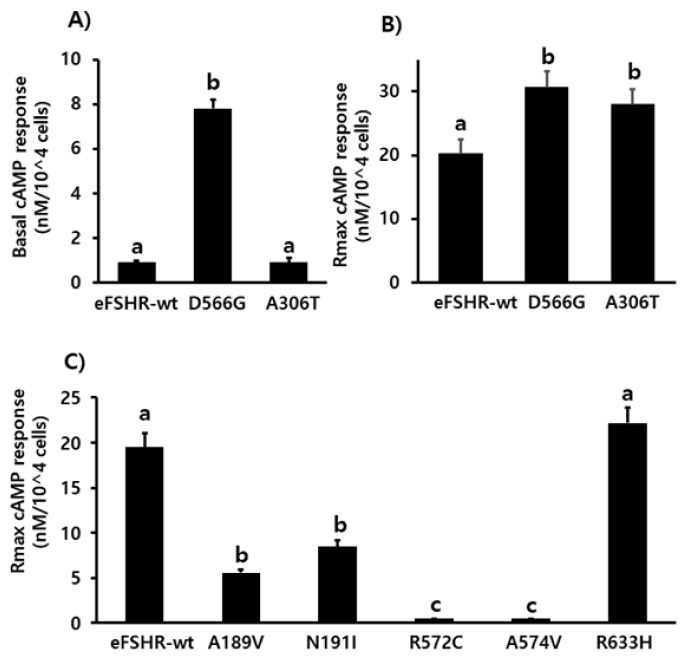
Determination of cAMP production in PathHunter parental CHO-K1 cells transiently expressing the wild-type and mutant eFSHRs. cAMP production in cells expressing the wild-type and mutant eFSHRs. (A) Basal accumulation in the absence of agonist treatment; (B) Rmax levels among eFSHR-wt and eFSHR mutants with activating mutation D566G and allelic variant A306T, and (C) Rmax levels among eFSHR-wt and eFSHR inactivating mutants. Data have been expressed as mean±standard error of the mean of triplicate experiments. eFSHR, equine follicle-stimulating hormone receptor. Values with different superscripts were significantly different (p<0.05).

The EC_50_ of eFSHR-A189V as well as eFSHR-R572 and -A574V was not estimated, while the EC_50_ of eFSHR-N191I was found to be approximately 5.4-fold (3,910 ng/mL) lower. The mutant eFSHR-R633H, carrying the mutation in the cytoplasmic tail, showed similar EC_50_ and Rmax levels as the wild-type receptor, an effect identified in case of the allelic variant mutant eFSHR-A306T as well (Figure 4).

## DISCUSSION

The present study analyzed allelic variants in eFSHR as well as mutations known to induce constitutively activating/inactivating signal transduction, in order to confirm if they have similar functionality as previously reported hFSHR mutations that are known to cause ODG, POF, and sOHSS. We constructed seven eFSHR mutants that are highly conserved among glycoprotein hormone receptors. These mutants either considerably stimulated basal cAMP accumulation in the absence of agonist treatment (eFSHR-D566G) or completely impaired agonist-induced activation of the receptor (eFSHR-A189V, -N191I, -R572C, and -A574V). The allelic variant mutant (eFSHR-A306T) and the inactivating mutant (eFSHR-R633H) showed effects that were similar to those of eFSHR-wt.

In the present study, eFSHR-D566G (known as the constitutively active mutant) showed remarkably increased basal cAMP responsiveness in the absence of agonist treatment. The chimeric receptor of FSHR-LHR (FL-VC), containing the D567G mutant and the portion of the LH receptor from the transmembrane domain V to the carboxyl terminus, led to constitutive activation of the receptor, with a 7.8-fold increase in basal cAMP response as compared to wild-type FL-VC [35]. A study showed that there is impaired post-translational processing of the 73-kDa form of the rLHR-D556G mutant receptor into the mature 92-kDa receptor at the Golgi, which prevents its trafficking to the cell surface [36]. Cells expressing rFSHR-3L, with a mutation of T/S to A in the third intracellular loop, exhibited high-basal cAMP responsiveness and an elevated maximal response to FSH [37]. Deletions in the intracellular loop connecting TM 5 and TM 6 of the FSHR resulted in drastically reduced membrane expression, indicating the importance of the intracellular loop for signal transduction [38]. These results are consistent with our data, showing that eFSHR-D566G is constitutively active in the absence of agonist treatment. Recently, we also reported that the eelFSHR-D540G mutant in fish produced a 23.2-fold increase in basal cAMP production [32]. Thus, we suggest that these activating sites, including eFSHR-D566G in FSHRs have the same functionality in terms of basal cAMP response. This may be a result of a change in FSHR conformation, caused by the amino acid residue transition. Recently, it was shown that transgenic mice expressing the hFSHR-D567G mutant gene in the Sertoli cells, displayed increased specific binding of FSH to the testis membrane and elevated constitutive cell signaling [39]. The FSHR in transgenic mice is probably not translated into the functional receptor protein [40], suggesting that the D576G mutation confers autonomous signaling and steroidogenic activity *in vivo* [41]. Thus, we believe that the activating sites of the third intracellular loop in FSHR seem to be essential for FSH-FSHR interaction and FSH-induced cAMP response. Further studies will need to be carried out to understand why these activating mutants cause an elevation in basal cAMP production and are common in patients with ODG, POF, and sOHSS.

The other activating mutants were reported in the rFSHR-D580G and hFSHR-D581G, suggesting that the D580G mutation in the TM 6 caused marked constitutive activation [10]. Substitution of hFSHR-L460 with K, A, or D suggested that only L460R mutant displayed constitutive activation [9]. The substitution of hFSHR-L460 and hLHR-L457 with R was reported the strongest constitutively activating mutants, suggesting that basal activity was very similar for remarkable structural similarities between hFSHR and hLHR [11]. In the present studies, we did not analyze the eFSHR-D580G and L459R mutants. Thus, further experiments are needed to explore the signal transduction for these two mutants.

Our results also demonstrated that the Rmax levels of the eFSHR-A306T mutant were 1.4-fold higher than those observed in case of the wild-type receptor, although the EC_50_ levels were almost similar. This polymorphism, along with the one at the 680th amino acid residue, were identified in patients with ODG, POF, and in infertile man [6,42,43]. Several other studies in different populations showed no such association with these diseases [12,17,44]. Recently, the polymorphisms A307T and S680N were reported in three out of four patients with ovarian juvenile granulosa cell tumors and in the three out of five controls [19]. hFSHR mutations do not play a pathogenic role in male idiopathic infertility, indicating similar binding affinities and cAMP responsiveness upon FSH treatment [1]. Amino acid sequence comparison results show that the position 306 is occupied by Ala in equine, bovine, ovine, and human sequences [45–47], whereas position 680 is occupied by Asn in these animals and by Ser in human. The single-strand conformation polymorphism was found to have a statistically significant difference in its genotypic distribution among the controls, POF, and diminished ovarian reserve groups; but no other variants were observed in hFSHR exon 10 [12]. Thus, our results in eFSHR-A306T are consistent with previously reported signal transduction research. This suggests that the eFSHR-A306T allelic variant has similar functionality in different mammalian species. However, it still remains to be understood why patients with this mutation suffer from infertility.

As predicted from the above results, the mutants analyzed in the present study (eFSHR-A189V, -N191I, -R572C, and -A574V) displayed impaired signal transduction, as previously reported in hFSHR [6,8,24,28]. It has been shown that the inactivating sites severely affect the signal transduction after FSH binding via a conformational change in FSHR that suppresses cell membrane targeting of FSHR [48]. Thus, our results are consistent with previously studies, which state that these inactivating mutations lead to a loss of FSHR function in cells expressing the mutant receptors. The conformational changes in the mutated receptor could explain why the inactivating mutants do not produce cAMP responses, despite of prolonged agonist stimulation. Specifically, the eFSHR-R633H is the only one to show an increase in cAMP response, similar to that seen in case of the wild-type receptor. However, these results are not consistent with a previous study that showed that hFSHR-R634H mutant displayed a lower response as compared to the wild-type receptor at high doses of FSH, but did not show any difference at low doses (1 to 5 mIU/mL) [27]. Among the inactivating mutants, eFSHR-R633H mutant can be assumed to be normally expressed on the cell surface. Previous studies have reported that constitutively activating FSHR and LHR mutants are usually quickly desensitization by phosphorylation and internalization [49]. Therefore, inactivating mutant exception eFSHR-R633H are assumed that it does not internalization by ligand. Thus, we suggest that studies on the eFSHR-R633H mutant are essential to reveal the signal transduction in cells expressing this mutant receptor.

In conclusion, this study was shown that the eFSHR-D556G, constitutively activating mutation-expressing receptor, displayed a significant increase in the basal cAMP production. The allelic variant-expressing mutant, eFSHR-A306T resulted in a higher maximal level as compared to the wild-type receptor, with no difference in the EC_50_ levels. Our data clearly showed that the four inactivating mutations (A191V, N191I, R572C, and A574V) completely impaired the signal transduction following FSH treatment. We suggest that eFSHR-A189V and N191I mutants result in a total loss-of-signaling. eFSHR-R572C and A574V showed completely impaired signaling in maximal cAMP responses. Interestingly, the mutant expressing eFSHR-R633H mutation, which is present at the cytoplasmic tail, showed a dose-dependent increase in the cAMP response, similar to that observed in case of the wild-type receptor.

Thus, we suggest that the two mutants eFSHR-A306T and eFSHR-R633H, which show high cAMP responsiveness, need to be focused upon when studying the cell surface expression and receptor internalization following FSH treatment. These results are indeed significant for our understanding of signal transduction involving mutations of highly conserved amino acids in mammalian FSH receptors. Future studies should aim to identify the mechanism responsible for the structure-function relationship of these activating/inactivating and allelic variant mutants.

## Figures and Tables

**Table 1:** Bioactivity of eFSH receptors in cells expressing wild-type as well as activating and allelic mutations

eFSH receptors	cAMP responses		

	Basal^1)^ (nM/10^4^ cells)	EC_50_^2)^ (ng/mL)	Rmax^3)^ (nM/10^4^ cells)
eFSHR-wt	0.9±0.1 (1-fold)	765.1 (599.7 to 1,056)^4)^	20.3±2.1 (1-fold)
eFSHR-D566G	7.8±0.4 (8.6-fold)	648.4 (543.7 to 803)	30.7±2.5 (1.5-fold)
eFSHR-A306T	0.9±0.2 (1-fold)	751.2 (609.7 to 978.1)	27.9±2.4 (1.4-fold)

Values are expressed as means±standard error of the mean of triplicate experiments.

The half maximal effective concentration (EC_50_) values were determined from the concentration-response curves of the *in vitro* bioassays. The basal cAMP responses and Rmax in wild-type eFSHR have been represented as 1-fold.

1) Average basal cAMP levels without agonist treatment.

2) Half maximal effective concentration.

3) Rmax average cAMP level/10^4^ cells.

4) Geometric mean (95% confidence limit).

**Table 2:** Bioactivity of eFSH receptors in cells expressing inactivating receptor mutations

eFSH receptors	cAMP responses		

	Basal^1)^ (nM/10^4^ cells)	EC_50_^2)^ (ng/mL)	Rmax^3)^ (nM/10^4^ cells)
eFSHR-wt	0.8±0.2	718.0 (589.7 to 917.4)^4)^	19.5±1.6 (1-fold)
eFSHR-A189V	0.6±0.1	-^5)^	5.6±0.3 (0.3-fold)
eFSHR-N191I	0.3±0.1	3,910 (3,532 to 4,378)	8.5±0.7 (0.4-fold)
eFSHR-R572C	0.3±0.1	-	-
eFSHR-A574V	0.2±0.1	-	-
eFSHR-R633H	0.3±0.1	1,160 (1,053 to 1,292)	22.2±1.7 (1.1-fold)

Values are expressed as mean±standard error of the mean of triplicate experiments.

EC50 values were determined from the concentration-response curves of the *in vitro* bioassays. Rmax cAMP response of the wild-type eFSHR has been represented as 1-fold.

1) Average basal cAMP levels without agonist treatment.

2) Half maximal effective concentration.

3) Rmax average cAMP level/10^4^ cells.

4) 95% confidence intervals.

5) Nondetectable.
